# Removal of Refractory Dissolved Organic Carbon in the Amundsen Sea, Antarctica

**DOI:** 10.1038/s41598-020-57870-6

**Published:** 2020-01-27

**Authors:** Ling Fang, SangHoon Lee, Shin-Ah Lee, Doshik Hahm, Guebuem Kim, Ellen R. M. Druffel, Jeomshik Hwang

**Affiliations:** 1 School of Earth and Environmental Sciences/Research Institute of Oceanography, Seoul National University, Seoul, 08826 South Korea; 2 Korea Polar Research Institute, Incheon, 21990 South Korea; 3 Pusan National University, Busan, 46241 South Korea; 4 Department of Earth System Science, University of California, Irvine, CA 92697 USA; 5 Present Address: Laboratory of Environmental Chemistry, Paul Scherrer Institute, Villigen, 5303 Switzerland

**Keywords:** Biogeochemistry, Ocean sciences

## Abstract

The removal mechanism of refractory deep-ocean dissolved organic carbon (deep-DOC) is poorly understood. The Amundsen Sea Polynya (ASP) serves as a natural test basin for assessing the fate of deep-DOC when it is supplied with a large amount of fresh-DOC and exposed to strong solar radiation during the polynya opening in austral summer. We measured the radiocarbon content of DOC in the water column on the western Amundsen shelf. The radiocarbon content of DOC in the surface water of the ASP reflected higher primary production than in the region covered by sea ice. The radiocarbon measurements of DOC, taken two years apart in the ASP, were different, suggesting rapid cycling of DOC. The increase in DOC concentration was less than expected from the observed increase in radiocarbon content from those at the greatest depths. Based on a radiocarbon mass balance, we show that deep-DOC is consumed along with fresh-DOC in the ASP. Our observations imply that water circulation through the surface layer, where fresh-DOC is produced, may play an important role in global DOC cycling.

## Introduction

The concentration of dissolved organic carbon (DOC) in the deep ocean (>1000 m, hereafter deep-DOC) is vertically uniform, but varies horizontally from ~34 to 48 μmol kg^−1^ (ref. ^1^). The ^14^C ages of deep-DOC range from ~4000 yr in the North Atlantic to ~6500 yr in the North Pacific^2–6^. The old ^14^C ages, together with its resistance to bacterial consumption^7^, imply that deep-DOC is refractory. A decrease in deep-DOC concentration of ~29% along the deep water circulation path is consistent with a very slow consumption rate^8^. Bacterial degradation, photochemical degradation, and adsorption to particles have been demonstrated as potential removal mechanisms^8–13^. However, the removal processes of deep-DOC are still not clearly understood.

**Figure 1:**
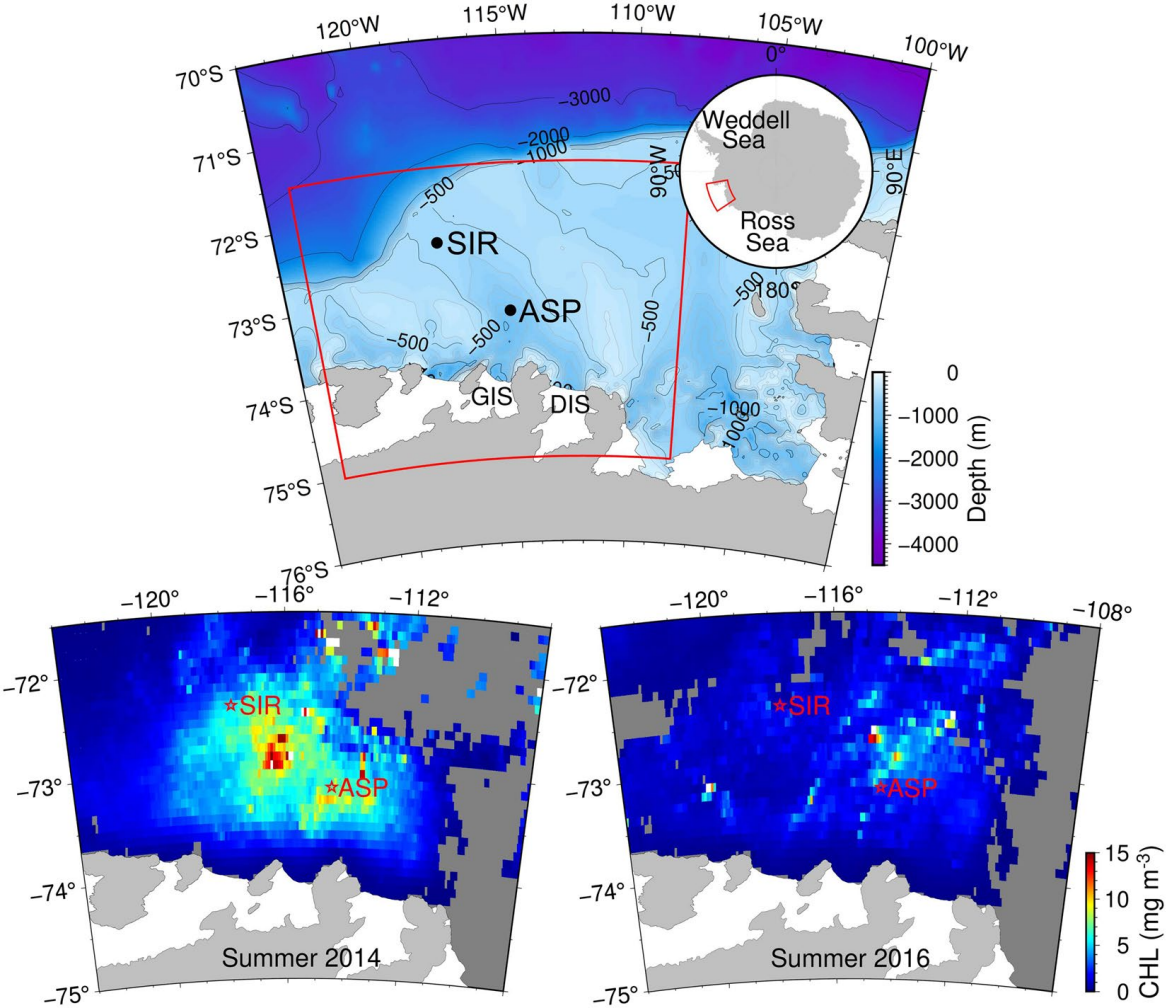
Bathymetry of the Amundsen Sea, showing the locations of the two sampling sites SIR and ASP (upper panel). DIS and GIS denote Dotson Ice Shelf and Getz Ice Shelf, respectively. The red box indicates the region for which chlorophyll-*a* data are shown in the lower panels. Satellite-based average chlorophyll-*a* concentrations in summer from November 2013 through February 2014 (lower left) and from November 2015 through February 2016 (lower right). The chlorophyll-*a* data are MODIS-Aqua Level 3 images (R2018.0) with temporal and spatial resolutions of 8 days and 0.083° by 0.083°, respectively (https://oceancolor.gsfc.nasa.gov/). Figures were produced using GMT 5.4.5 (http://gmt.soest.hawaii.edu/).

The Amundsen Sea in the west Antarctic is characterized by a relatively broad and deep continental shelf and extensive perennial sea ice cover^14^ (Fig. 1). Circumpolar Deep Water (CDW) flows onto the Amundsen Shelf along the seafloor^15–17^. A part of the intruding CDW mixes with the overlying water to form modified CDW (MCDW). In the surface layer above ~400 m, water flows north, northwest, and off the shelf in general^18,19^. The western Amundsen Sea harbors a highly productive polynya, the Amundsen Sea Polynya (ASP)^20–22^. Peak annual production can be as high as ~3 gC m^−2^d^−1^ with an average annual production of 105 gC m^−2^yr^−1^ (ref. ^22^). High primary production in summer supplies considerable DOC (fresh-DOC) to the euphotic layer^20^. Deep-DOC supplied to the surface layer of the Amundsen Sea via CDW intrusion and mixing with the overlying water is subjected to biogeochemical processes such as the addition of extensive fresh-DOC^20^ and microbial^23^ and/or photochemical degradation^10^. The turnover time of the waters in the western Amundsen Sea embayment was suggested to be a few decades based on the ^14^C content of dissolved inorganic carbon (DIC)^24^. Because of this unique environmental setting^25^, the ASP can be considered a natural test basin, acting as a laboratory incubation vessel, for assessing the fate of deep-DOC once it is brought into a shallower environment. We take advantage of this environmental setting to investigate DOC cycling with a focus on the fate of deep-DOC.

## Results and Discussion

### Hydrography

DOC samples were collected during two cruises in the western Amundsen Sea in January 2014 (the ANA04B cruise) and in January 2016 (the ANA06B cruise). The two sampling sites had different surface water conditions: one was in the sea-ice-covered region (SIR) north of the ASP and the other in the central region of the ASP (Fig. 1). For convenience, we refer to the stations as SIR-2014 and ASP-2014 (2014 cruise), and SIR-2016 and ASP-2016 (2016 cruise).

**Figure 2:**
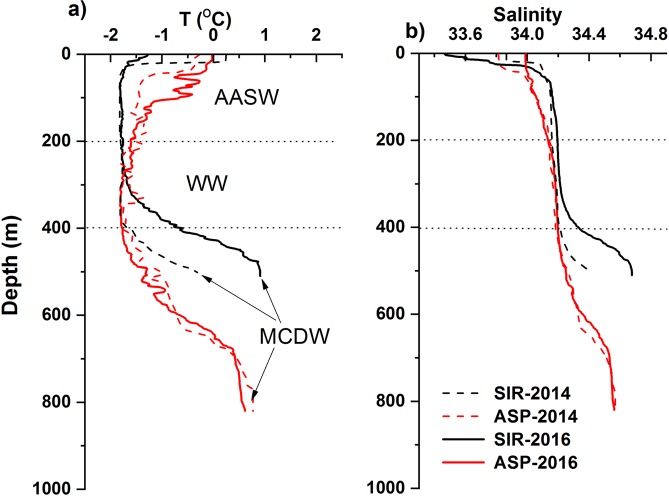
Vertical profiles of (**a**) potential temperature and (**b**) salinity at the stations in 2014 and 2016. The arrows indicate MCDW. AASW = Antarctic Surface Water; WW = Winter Water; MCDW = Modified Circumpolar Deep Water.

The warmest and most saline MCDW occupied the greatest depths at both sites (Fig. 2). The highest temperature of this layer was lower than that of CDW (>1 °C)^26^, indicating that the characteristics of CDW were modified by mixing with overlying Winter Water (WW) on the shelf. Based on salinity as a conservative property in the deep layer, MCDW at the greatest depths contained 57%, 24%, 2%, and 25% of WW at SIR-2014, ASP-2014, SIR-2016, and ASP-2016, respectively. The potential temperature and salinity of MCDW observed in 2014 and 2016 at the ASP site were virtually identical. However, both potential temperature and salinity were different at the SIR site, showing greater modification of MCDW in 2014 than in 2016. WW of the lowest temperature with varying thickness occupied the middle layer. The temperature and salinity of the layer between WW and MCDW reflected mixing of the two water masses. Above the WW layer, Antarctic Surface Water (AASW) with elevated temperature and lower salinity occupied the surface layer. The surface mixed layer was shallow (~30 m) at the SIR site, while it was deeper at the ASP site, especially at ASP-2016, when seasonal warming of the surface water reached ~300 m.

### Distribution of DOC concentration and radiocarbon (∆^14^C)

**Figure 3:**
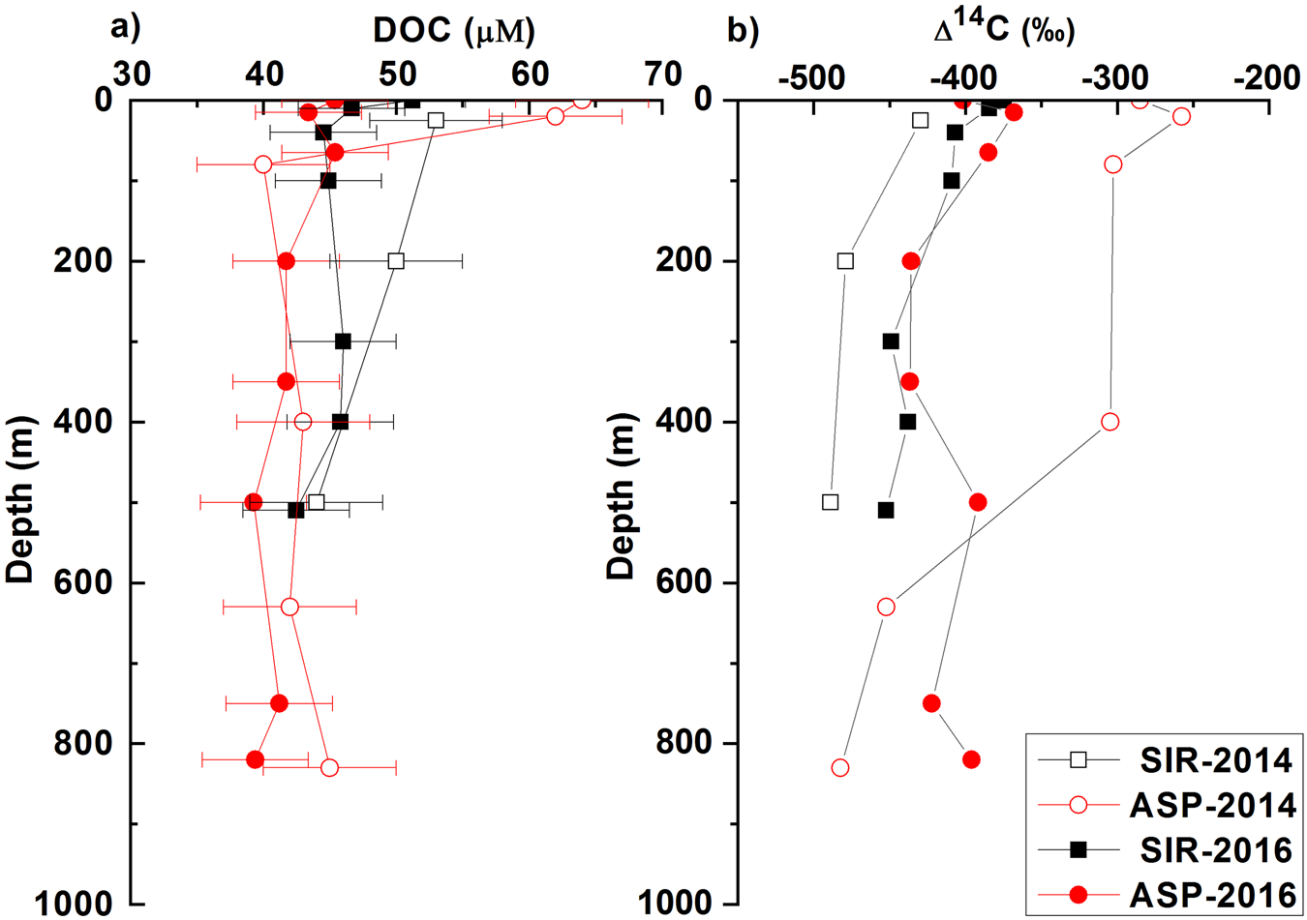
Vertical profiles of (**a**) concentration and (**b**) Δ^14^C values of dissolved organic carbon (DOC).

DOC concentrations in the surface water ranged between 45 and 64 μM and were in general higher than the values at deeper depths. Below ~100 m, DOC concentration ranged between 39 and 50 μM with an average of 43 ± 3 μM (Fig. 3a). The slightly increased value (50 μM) observed at 200 m may be an outlier. DOC concentrations near the seafloor in all cases (42 ± 2 μM) were virtually identical to those observed for CDW (at depths > 1000 m) in the Southern Ocean (41 μM at 54°S, 176°W; 39 μM at 55.6°S, 84.7°E)^27,28^.

Production of DOC in the surface water was evident especially at ASP-2014. The difference in DOC concentration between the SIR and ASP sites in 2014 was consistent with the difference in primary production. In 2014, our sampling occurred during the peak bloom, and the ASP site was within the region of high chlorophyll-*a* concentration, as indicated by satellite images of chlorophyll-*a* distribution (Fig. 1 and Supplementary Fig. S1). During the bloom in the ASP, primary production supplies a large amount of DOC to the euphotic layer^20^. Dissolved oxygen was supersaturated by 8% in the surface water at ASP-2014, a sign of high photosynthetic activity (Supplementary Fig. S2). However, in 2016 the phytoplankton bloom started farther northeast of the polynya and our ASP site was not yet fully affected by the bloom at the time of sampling (Fig. 1 and Supplementary Fig. S1). Dissolved oxygen was slightly undersaturated in the surface water at ASP-2016.

During the two cruises, Δ^14^C values of DOC ranged between −489‰ and −257‰ (Fig. 3b). The Δ^14^C values in the surface water were higher than those in deeper water, and lowest in the deep water, with the exception of ASP-2016. These lowest values are similar to the Δ^14^C values observed previously for CDW^27,28^. The Δ^14^C values of the deepest sample from SIR-2014 (−489‰) and SIR-2016 (−452‰) were significantly different (Student’s *t* test, *p = *0.04, *t = *−2.3), although the corresponding DOC concentrations were similar. This difference in Δ^14^C values of the deepest samples between the two years could be partly introduced by varying Δ^14^C of the CDW source over the two years. However, published Δ^14^C data of CDW^27,28^ observed in different years (1995 and 2015) and locations were identical, suggesting that the DOC Δ^14^C value of CDW likely remained constant. Alternatively, higher Δ^14^C values in 2016 than in 2014 near the seafloor may have been the result of penetration of freshly produced DOC into the sea interior by sinking of particulate organic matter and vertical mixing of water masses. Biological processes such as primary productivity and microbial consumption of DOC in previous years can be important for the distribution of fresh-DOC in the water column. The observed difference in Δ^14^C between the two years implies that DOC cycling was dynamic.

**Figure 4:**
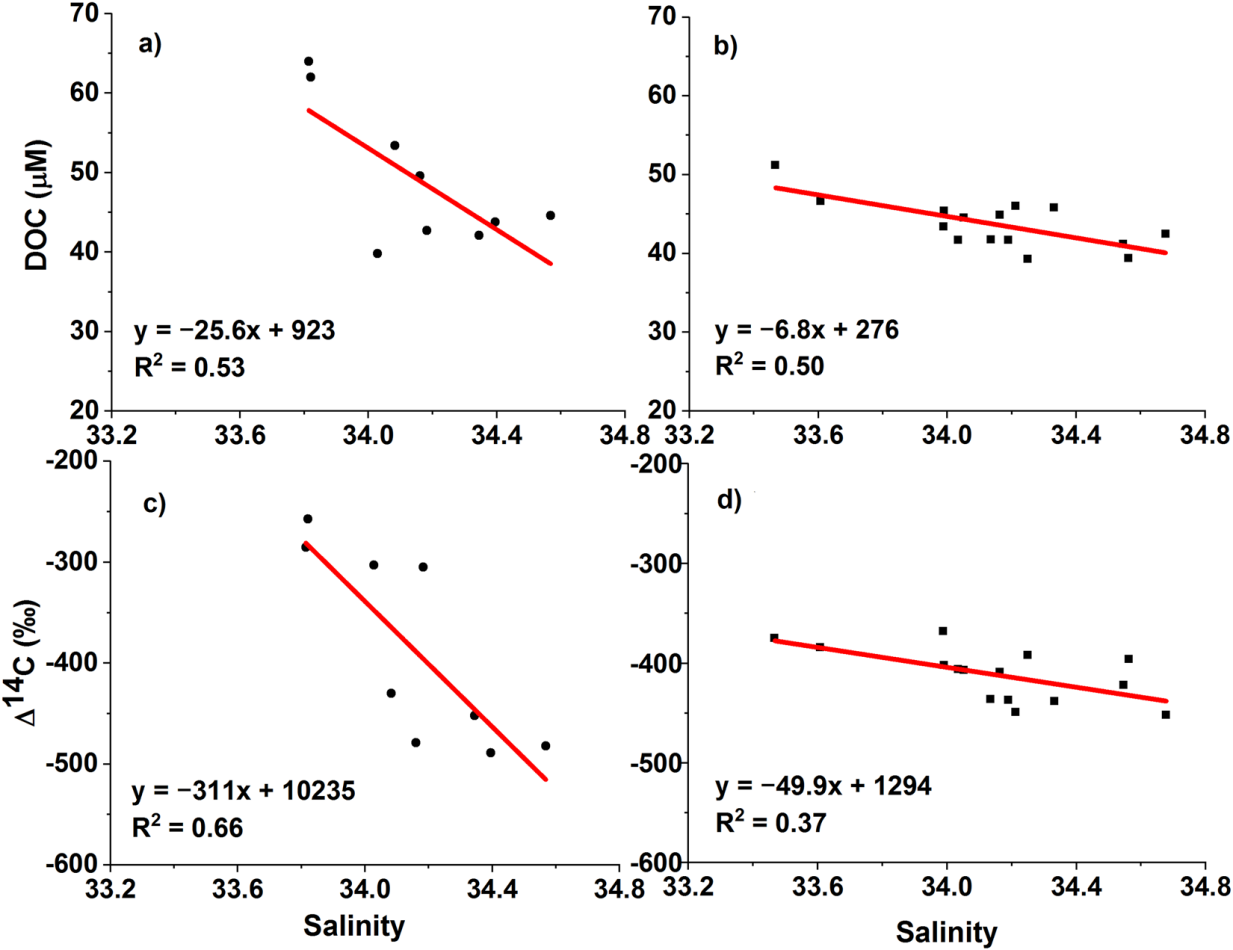
DOC concentration versus salinity in (**a**) 2014 and (**b**) 2016. Δ^14^C value versus salinity in (**c**) 2014 and (**d**) 2016.

DOC concentration and Δ^14^C values at both sites exhibited negative correlations with salinity in both 2014 and 2016 (Fig. 4). These correlations imply that the distribution of DOC concentration and radiocarbon values in the water column is controlled primarily by vertical mixing of water masses. The slopes of the linear regressions were significantly steeper in 2014 than in 2016, although they were strongly influenced by high values of DOC concentration and ∆^14^C in the surface waters (Fig. 4).

### DOC cycling in the Amundsen Sea

Previous studies have reported that DOC concentrations and Δ^14^C values show a positive correlation at a given site in the ocean^4,29,30^. In the open ocean, this correlation can be explained by mixing between two components, background DOC (DOC_bg_) and DOC added in excess (DOC_xs_), where DOC_bg_ concentration remains unchanged. Additional sources of DOC with Δ^14^C values different from that of the DOC_xs_ would disrupt the correlation. Our current understanding of water circulation in the western Amundsen Sea embayment is that CDW inflows along the seafloor, vertical mixing occurs with the overlying water, and water flows out of the shelf in the upper ~400 m layer^18,19^. Horizontal mixing in the study region is assumed to be negligible. We examined the effect of DOC from glacial melting as a potential source of allochthonous DOC, considering that meltwater content in seawater is 0.3–0.8% along the Dotson Trough, a glacier-carved trough in the western Amundsen Sea^31^. DOC supply from glacier meltwater with a concentration of 33 μM^32^ and Δ^14^C values of −600‰ to modern^33^ may change the DOC concentration by <0.5 μM and Δ^14^C values by <5‰.

We applied a Keeling mixing model to test whether the observed DOC concentration and Δ^14^C values can be explained by the simple addition of fresh-DOC to deep-DOC. A plot of Δ^14^C vs. 1/[DOC], where [DOC] denotes the DOC concentration^29^, would show a linear trend in the case that DOC_xs_ is added to DOC_bg_ and DOC_bg_ remains unchanged. Note that mixing between different components of DOC can occur independently of water mass mixing. Addition/consumption of DOC can occur in a given water mass without changing conservative properties. Also, mixing between water masses (ASW, WW, and MCDW) in the water column would not change the correlation as long as the DOC_bg_ concentration remains constant. The plots show a significant linear trend only at SIR-2016 (R^2^ = 0.73; Supplementary Fig. S3). The *y*-intercept value, −121 ± 72‰, is similar to the Δ^14^C value of DIC in the surface water (−129‰ to −162‰)^24^, implying that *in situ* primary production was the main source of fresh-DOC at this site. At the other sites, either the plot did not show any significant linear trend (R^2^ < 0.3) or the uncertainty of the intercept (~±200‰) was too large to provide meaningful information. Because no significant allochthonous source of DOC was identified in the study region, the insignificant correlations imply that consumption of deep-DOC occurs (therefore, the DOC_bg_ concentration changes).

**Figure 5:**
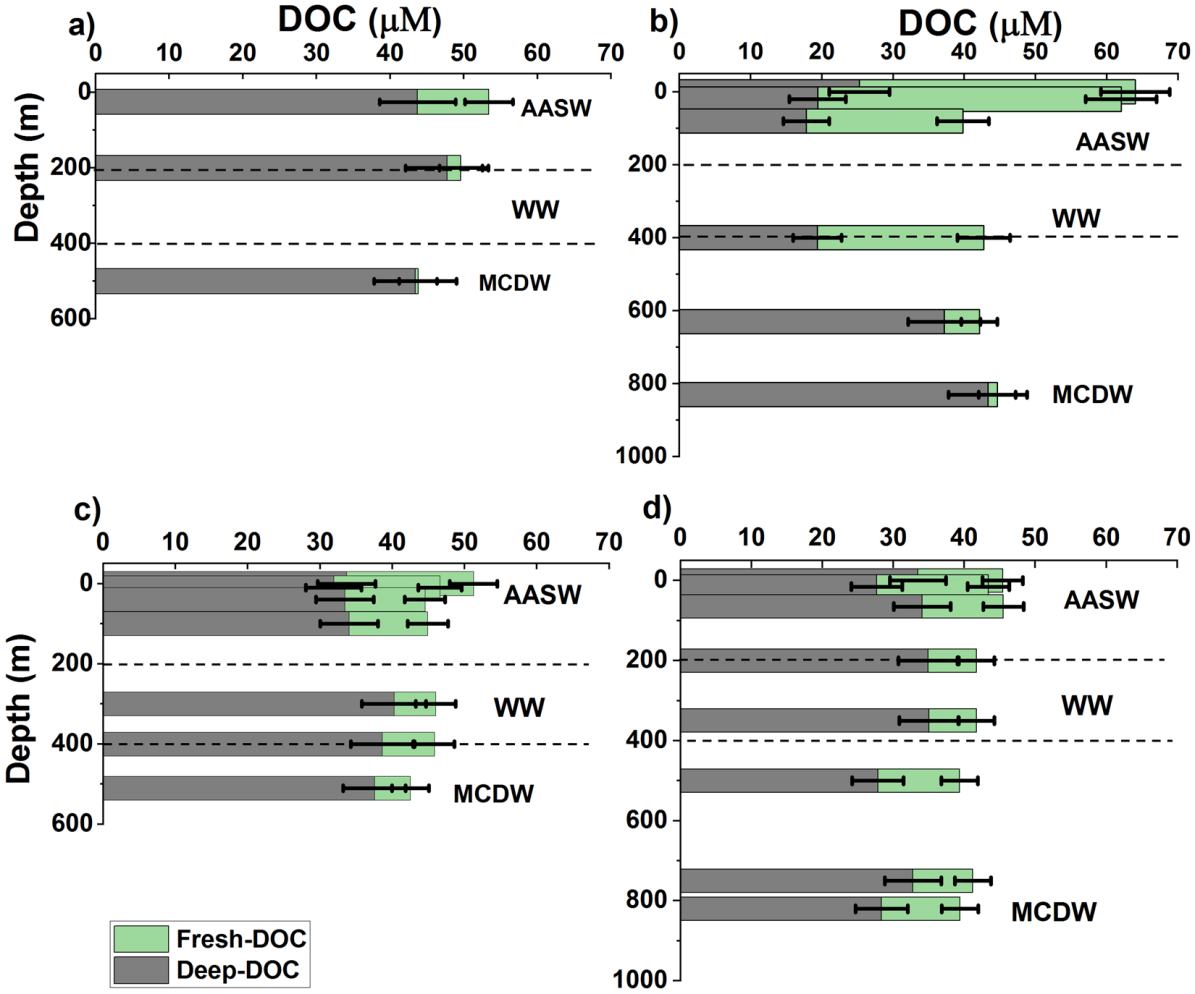
Vertical distribution of fresh-DOC and deep-DOC based on a radiocarbon mass balance for (**a**) SIR-2014, (**b**) ASP-2014, (**c**) SIR-2016, and (**d**) ASP-2016. Error bars indicate total uncertainty derived from DOC concentration and radiocarbon ∆^14^C measurements.

Concentrations of fresh-DOC and deep-DOC can be estimated by a radiocarbon mass balance^34^. In this way, the magnitude of deep-DOC consumption in the water column can be calculated. We estimated the fractions of fresh-DOC and deep-DOC as follows:

1 $${[{\rm{DOC}}]}_{{\rm{observed}}}=[{\rm{deep}} \mbox{-} {\rm{DOC}}]+[{\rm{fresh}} \mbox{-} {\rm{DOC}}]$$

2 $${[{\rm{DOC}}]}_{{\rm{observed}}}\times {\Delta }^{14}{{\rm{C}}}_{{\rm{observed}}}=[{\rm{deep}} \mbox{-} {\rm{DOC}}]\times {\Delta }^{14}{{\rm{C}}}_{{\rm{deep}}}+[{\rm{fresh}} \mbox{-} {\rm{DOC}}]\times {\Delta }^{14}{{\rm{C}}}_{{\rm{fresh}}}$$

where Δ^14^C_deep_ = −492 ± 20‰ (mean value of CDW previously reported at 54°S, 176°W and 55.6°S, 84.7°E^25,26^) and Δ^14^C_fresh_ = −149 ± 10‰ from surface water DIC values^24^. Taking the surface sample at ASP-2014 with a Δ^14^C value of −285‰ as an example, we calculated that it contained 39% deep-DOC (25 ± 4 μM) and 61% fresh-DOC (39 ± 5 μM). In the water column at ASP-2014, the concentration of deep-DOC generally decreased from the bottom to the surface rather than staying constant (Fig. 5). The estimated concentration of deep-DOC (25 ± 4 μM) in the surface water is much lower than the observed DOC concentration near the seafloor (42 ± 2 μM). We suggest that this discrepancy in the deep-DOC concentration in the water column represents the portion of deep-DOC that has been removed. The concentration of fresh-DOC increased from the bottom to the surface (Fig. 5b). Fresh-DOC appears to be mostly confined within WW and AASW. However, at ASP-2016, fresh-DOC was present near the seafloor as well, presumably owing to vertical mixing of MCDW and WW.

In this calculation, we assumed that deep-DOC was homogeneous and therefore its Δ^14^C value did not change during its consumption^5^. Follett *et al*.^35^ showed that deep-DOC contains modern DOC as well. Therefore, our assumption would result in underestimation of deep-DOC consumption if a younger component of deep-DOC was preferentially consumed.

The processes identified by Shen and Benner^13^, namely priming (addition of labile substrates), exposure to coastal microbes, photodegradation, and photo-enhanced biodegradation, are all applicable in the Amundsen Sea, especially during the polynya opening. Consumption of deep-DOC was most prominent at ASP-2014. At ASP-2016, primary production was much lower than in 2014 (Fig. 1 and Supplementary Fig. S1). Our sampling was performed in January during both cruises. These observations suggest that the supply of fresh-DOC can be potentially more important than sunlight availability for removal of deep-DOC. However, the importance of light cannot be ruled out, because primary productivity itself may be related to sunlight availability, as suggested by Park *et al*.^36^. In the SIR, removal of deep-DOC was low during both years, probably because of both UV reduction by sea ice and lower primary production.

Based on our snapshot observations during two years, we describe DOC cycling in the ASP as follows. During the phytoplankton bloom in summer, a large amount of fresh-DOC is added and refractory deep-DOC is removed (as recorded at ASP-2014). DOC is redistributed vertically by water mixing in winter^37^, transporting fresh-DOC to great depths (as recorded at ASP-2016). The difference in distribution of fresh-DOC and deep-DOC in the ASP between 2014 and 2016 implies that the DOC reservoir has a relatively short turnover time on the order of years.

### Implications for global deep-DOC cycling

Based on the radiocarbon data of DOC in the western Amundsen Sea, we show that deep-DOC can be consumed on the continental shelf, especially when a large amount of fresh-DOC is supplied from high primary production during the polynya opening in summer. Our observations provide field evidence for the removal of deep-DOC in the natural environment, as had been demonstrated by laboratory experiments^13^. Similar processes are expected to occur in the western Antarctic where CDW intrudes onto the shelf, and in other productive Antarctic coastal polynyas. Well-known regions of upwelling, such as the eastern Equatorial Pacific and the western coasts of North and South America, have potential for deep-DOC consumption. Therefore, water circulation through the surface layer, where fresh-DOC is produced, may play an important role in global DOC cycling and removal of deep-DOC.

## Methods

Water samples for measurements of DOC concentration and radiocarbon isotope ratio were collected using a rosette system equipped with Niskin bottles. Seawater was drained into a sampling bottle through 0.7 μm Whatman quartz filters that were prebaked at 450 °C for 4 h. Amber glass sampling bottles (500 ml) were pre-cleaned with 10% HCl, rinsed with deionized water, and baked at 450 °C for 4 h. Samples were stored frozen at −20 °C in the dark until analysis. The ANA04B (SIR-2014 and ASP-2014) and ANA06B (SIR-2016 and ASP-2016) samples were analyzed in October 2015 and March 2016, respectively.

For radiocarbon analysis, DOC was converted to CO_2_ by irradiation with high-energy UV^38^. Each thawed sample was poured into a quartz reactor and acidified to pH 2–3 by addition of 85% H_3_PO_4_. Then the sample was sparged with high-purity nitrogen gas (99.999%) with a flow rate of 200 ml min^−1^ for ~60 min until the concentration of CO_2_ measured by an NDIR (non-dispersive infrared) CO_2_ sensor had decreased to the background value. The sample was then irradiated with UV (1200 W) for 6 h. At the end of irradiation, converted CO_2_ was recovered by N_2_ gas sparging. Recovered CO_2_ was cryogenically purified and quantified by pressure measurement, then stored in a Pyrex tube. The average recoveries of glucose and glycine standards were +90 ± 5% (*n* = 10) and +96 ± 1% (*n* = 2; the ± value indicates the range of the results when *n* = 2), respectively. Oxalic acid (OX-I) and glycine were used as process standards. Radiocarbon measurements of the standards were performed at the Keck Carbon Cycle Accelerator Mass Spectrometer Facility at UC Irvine, California USA. The Δ^14^C value of processed OX-I standard was +13‰ (*n* = 2) and that of glycine was −970‰ (*n* = 2). The true Δ^14^C values of the oxalic acid standard and glycine, +31‰ and −1000‰^39^, respectively, were used for blank correction. Based on a mass balance approach^40^, the blank amount was estimated to be 1.9 ± 0.2 μmol C with a Δ^14^C value of −330‰. The blank Δ^14^C value indicates that the blank was a mixture of carbon from the modern atmosphere and petroleum products such as O-rings and grease in the extraction device. Because the Δ^14^C value of the blank is similar to those of the samples, the blank correction was <27‰. Stable carbon isotope ratios (δ^13^C) of the samples were determined at the National Ocean Sciences Accelerator Mass Spectrometry Facility (NOSAMS) at the Woods Hole Oceanographic Institution^41^. All radiocarbon isotope ratio results were blank-corrected. We assigned uncertainties of ±10‰ for Δ^14^C, based on duplicate analyses of standards. Our δ^13^C values of samples were lower than typical marine values by a few per mil. In the Amundsen Sea, low δ^13^C values (−24.8‰ to −28.4‰) were observed for suspended particulate organic carbon (POC) in surface waters because of higher isotopic fractionation at low temperature^42^. Even taking these low values of POC into account as a source of fresh-DOC, the DOC δ^13^C values appear too low. Isotopic fractionation during the DOC extraction process cannot be ruled out and therefore we refrain from interpretation of the observed δ^13^C values. There is no consensus about the correction method for blank incorporation or isotopic fractionation for δ^13^C measurements^39^. However, isotopic fractionation, if it occurred, would not have affected the Δ^14^C values because they are fractionation-corrected values^43^.

For ANA06B samples, a portion (~50 ml) was subsampled upon thawing of each sample, and was analyzed for DOC concentration using a total organic carbon (TOC) analyzer (TOC-VCPH, Shimadzu) with an uncertainty of 2 μM^44^. Recovery of these samples at the end of UV irradiation was 101 ± 8% (*n* = 21, Supplementary Fig. S4). DOC concentrations for ANA04B samples were not measured and were instead estimated from the amount of CO_2_ recovered at the end of UV irradiation and the average recovery of the ANA06B samples. We assigned 5 μM as the uncertainty for DOC concentrations of ANA04B samples based on the standard deviation of our recovery.

## Supplementary information

Supplementary information.
