# Supplementary material for: Removal of Refractory Dissolved Organic Carbon in the Amundsen Sea, Antarctica
Source: Sci Rep. 2020 Jan 27;10:1213. doi: 10.1038/s41598-020-57870-6 (PMC6985272; doi:10.1038/s41598-020-57870-6)
Supplement: Supplementary file 1 — Supplementary information. [file 41598_2020_57870_MOESM1_ESM.pdf]

Scientific Reports

Supporting Information for

Removal of Refractory Dissolved Organic Carbon in the Amundsen Sea, Antarctica

Fang Ling[1,†], SangHoon Lee[2], Shin-Ah Lee[1], Doshik Hahm[3], Guebuem Kim[1], Ellen R. M. Druffel[4], Jeomshik Hwang[1]

[1]School of Earth and Environmental Sciences/Research Institute of Oceanography, Seoul National University, Seoul 08826, South Korea.

[2]Korea Polar Research Institute, Incheon 21990, South Korea.

[3]Pusan National University, Busan 46241, South Korea.

[4]Department of Earth System Science, University of California, Irvine, CA 92697, USA.

*Corresponding author: Jeomshik Hwang (jeomshik@snu.ac.kr)

†Current address: Laboratory of Environmental chemistry, Paul Scherrer Institute, Villigen 5232, Switzerland.

Contents of this file

Figures S1 to S4
Table S1

Supporting Information

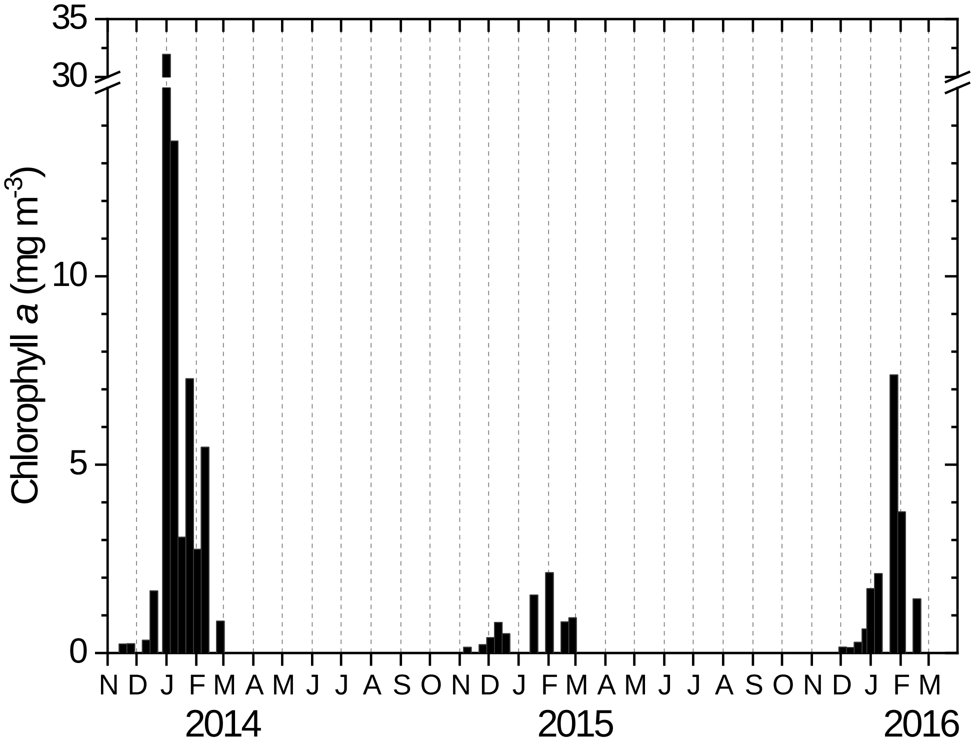

Figure S1. Temporal variations in chlorophyll *a* concentration at the ASP site. The satellite-observed chlorophyll *a* concentrations were averaged for the area around Station ASP (within 10 km radius). The concentrations were extracted from MODIS-Aqua level 3 chlorophyll *a* images with temporal and spatial resolutions of 8 days and 0.0083° × 0.0083°, respectively. Gaps indicate no data.

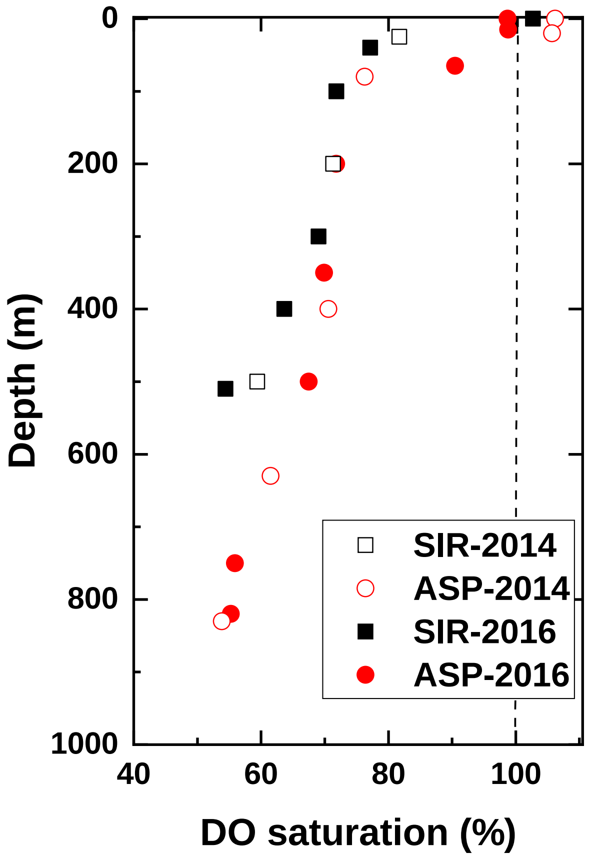

Figure S2. Dissolved oxygen (DO) saturation at sampling depths. DO saturation was calculated from oxygen concentration, salinity, and temperature using the equation from Garcia and Gordon [1] based on the values from Benson and Krause[2].

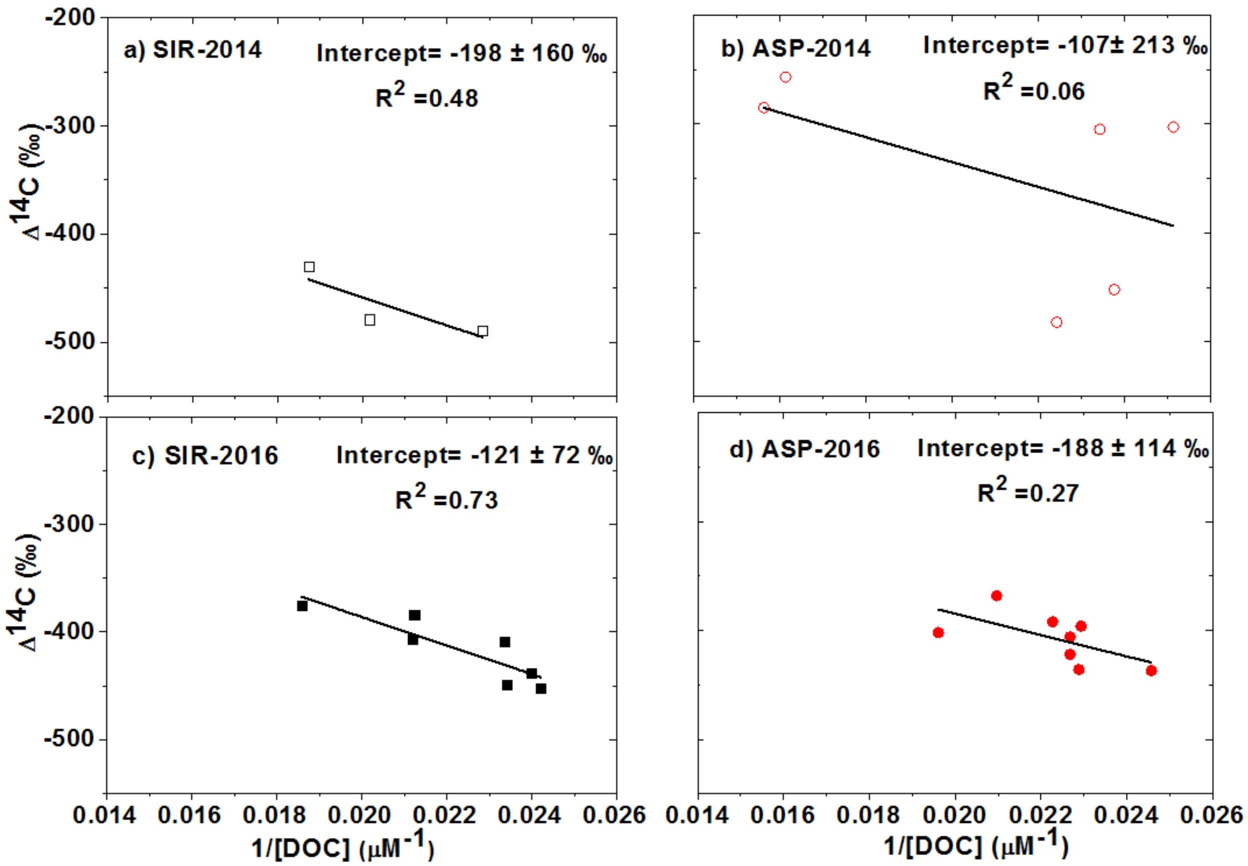

Figure S3. Keeling plots of the four cases.

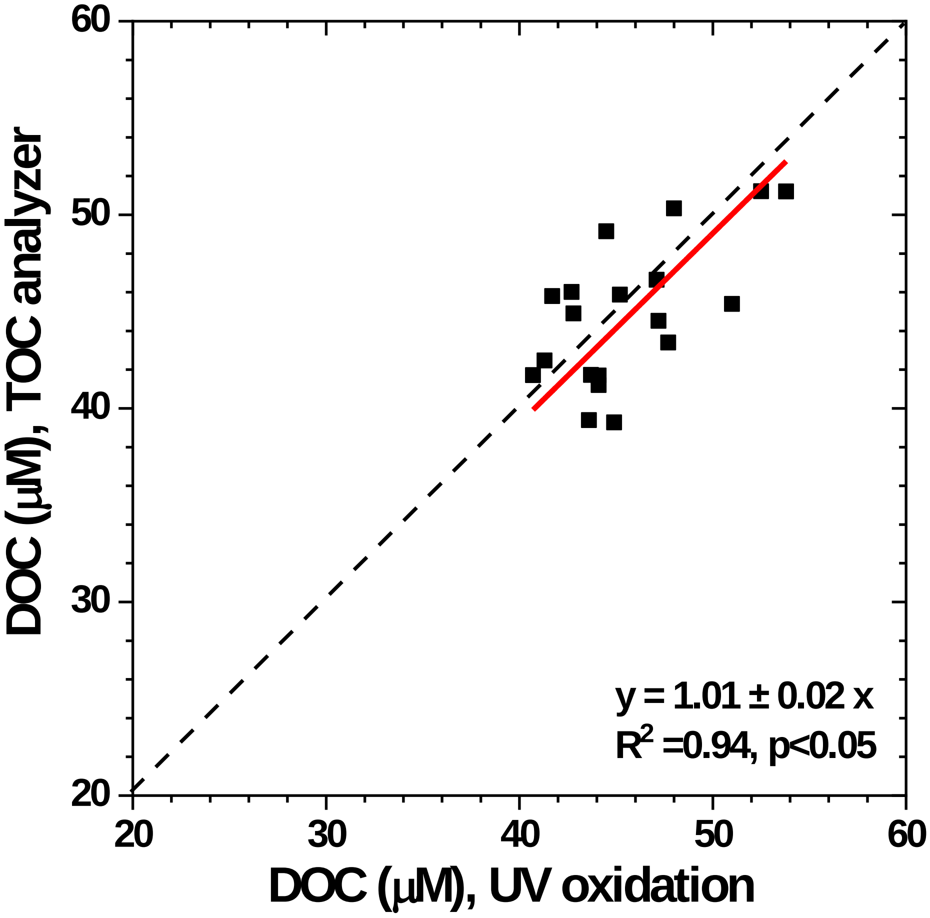

Figure S4. DOC concentrations (μM) measured using a TOC analyzer versus using UV oxidation. Regression fitting is showed as the red line in the figure.

Table S1. Concentrations and isotope values of DOC.

Station	Lat. (°S)	Lon. (°W)	Depth (m)	[DOC] (μM)	$\Delta^{14}C$ (‰) blank-corrected	$\delta^{13}C$ (‰) uncorrected
SIR-2014	72.39	117.71	25	53.4	-430	-26.1
			200	49.6	-479	-25.8
			500	43.8	-489	-27.7
ASP-2014	73.28	114.95	0	64	-285	-28.6
			20	62	-257	-28.3
			80	39.8	-303	-27.8
			400	42.7	-305	-27.2
			630	42.1	-452	-26.4
			830	44.6	-482	-25.9
SIR-2016	72.38	117.75	0	no data	-342	-25.5
			0	51.2	-375	-25.0
			10	46.6	-384	-23.6
			40	44.5	-407	-25.3
			100	44.9	-409	-24.8
			300	46.0	-449	-24.2
			400	45.8	-438	no data
			510	42.5	-452	-25.1
ASP-2016	73.28	114.95	0	45.4	-402	-25.4
			15	43.4	-368	-25.2
			65	49.2	-406	-25.2
			65	41.7	-385	-24.9
			200	41.7	-436	-25.7
			350	41.7	-437	-25.4
			500	39.3	-392	-25.3
			750	41.2	-422	-25.8
			820	39.4	-396	-24.9

References:

1. Garcia, H. E., and Gordon, L. I., Oxygen solubility in seawater: Better fitting equations. *Limnol. Oceanogr.* 37, 1307-1312(1992).

2. Benson, B. B., and Krause Jr, D., The concentration and isotopic fractionation of oxygen dissolved in freshwater and seawater in equilibrium with the atmosphere 1. *Limnol. Oceanogr.* 29, 620-632(1984).
